# Supplementary material for: Promising Results of Associating Liver Partition and Portal Vein Ligation for Staged Hepatectomy for Perihilar Cholangiocarcinoma in a Systematic Review and Single-Arm Meta-Analysis
Source: Cancers (Basel). 2024 Feb 13;16(4):771. doi: 10.3390/cancers16040771 (PMC10887221; doi:10.3390/cancers16040771)
Supplement: Supplementary file 1 [file cancers-16-00771-s001.zip › cancers-2822202-supplementary.pdf]

Promising Results of Associating Liver Partition and Portal Vein Ligation for Staged Hepatectomy for Perihilar Cholangiocarcinoma in a Systematic Review and Single-Arm Meta-Analysis

Supplementary Table S1. ALPPS indication, PHLF definition and cause of mortality in included studies.

Authors	Indication	PHLF	Grade A	Grade B	Grade C	Mortality
Schnitzbauer 2012 [7]	FLR/BW ratio < 0.5	Not reported		Not reported		-
Alvarez 2013 [10]	FLR < 30%	ISGLS		Not reported		-
Nadalin 2014 [22]	FLR <25% or FLR/BW ratio <0.5	Not reported		Not reported		Septic shock and MOF (n=1); 1 PHLF (n=1)
Ratti 2014 [23]	FLR after PVE <25% (normal parenchyma) or <40% (in other cases)	50-50 Balzan		Not reported		Septic shock and MOF (n=1)
Vivarelli 2015 [25]	FLR<30%	50-50 Balzan		Not reported		PHLF (n=1)
Kremer 2016 [24]	Not reported	ISGLS		Not reported		ND
Rosok 2016 [26]	FLR/BW ratio <0.5% or FLR <30% for patients with CRLM and FLR <40% in cases of suspected or confirmed cirrhosis	Not reported		Not reported		-

Serenari 2016 [27]	Not reported	ISGLS		Not reported		Septic shock (n=2), Cardiogenic shock (n=1)
Sakamoto 2018 [28]	FLR<30%	Not reported		Not reported		-
Kumar 2019 [29]	FLR<20-30% for normal liver and FLR <40% in cases with compromised liver	Not reported		Not reported		-
Balci 2020 [30]	FLR<30%	Not reported		Not reported		-
Melekhina 2020 [31]	FLR<40%	ISGLS	0	1	0	-
Chebaro 2021 [32]				Not reported		
Hotineanu 2021 [33]	FLR<30%	Not reported		Not reported		-
Stavrou 2022 [34]				Not reported		
Steffani 2022 [35]	FLR<40%	ISGLS		Not reported		-
Balci 2023 [21]	FLR < 30% or FLR/BW ratio <0.5	ISGLS	9	5	6	PHLF (n=1), Bile leak with PHLF (n=1), Bile leak with pneumonia (n=1)
Mehrabi 2023 [20]	FLR<30%	ISGLS	0	0	2	PHLF with MOF (n=2), Septic shock (n=2), Necrotizing pancreatitis (n=1)

ALPPS: Associating Liver Partition and Portal vein Ligation for Staged hepatectomy; BW: Body weight; CRLM: Colorectal liver metastases; FLR: Future remnant volume; ISGLS: International Study Group of Liver Surgery; MOF: Multiple organ failure; PHLF: Posthepatectomy liver failure; PVE: Portal vein embolization.
